# Role of Renal Sympathetic Nerve Activity in Volatile Anesthesia's Effect on Renal Excretory Function

**DOI:** 10.1093/function/zqab042

**Published:** 2021-08-20

**Authors:** Micael Taavo, Mats Rundgren, Peter Frykholm, Anders Larsson, Stephanie Franzén, Karin Vargmar, Jean F Valarcher, Gerald F DiBona, Robert Frithiof

**Affiliations:** Department of Surgical Sciences, Anesthesiology and Intensive Care, Uppsala University, Uppsala, Sweden; Department of Physiology and Pharmacology, Karolinska Institute, Stockholm, Sweden; Department of Surgical Sciences, Anesthesiology and Intensive Care, Uppsala University, Uppsala, Sweden; Department of Medical Sciences and Clinical Chemistry, Uppsala University, Uppsala, Sweden; Department of Surgical Sciences, Anesthesiology and Intensive Care, Uppsala University, Uppsala, Sweden; Department of Biomedical Sciences and Veterinary Public Health, Section of Pathology, Swedish University of Agricultural Sciences, Uppsala, Sweden; Department of Clinical Sciences, Division of Ruminant Medicine, Swedish University of Agricultural Sciences, Uppsala, Sweden; Carver College of Medicine, University of Iowa, Iowa, IA, USA; Department of Surgical Sciences, Anesthesiology and Intensive Care, Uppsala University, Uppsala, Sweden

**Keywords:** acute Kidney Injury (AKI), anesthesia, oliguria, urine output, ischemia, surgery, renal failure, kidney

## Abstract

Regulation of fluid balance is pivotal during surgery and anesthesia and affects patient morbidity, mortality, and hospital length of stay. Retention of sodium and water is known to occur during surgery but the mechanisms are poorly defined. In this study, we explore how the volatile anesthetic sevoflurane influences renal function by affecting renal sympathetic nerve activity (RSNA). Our results demonstrate that sevoflurane induces renal sodium and water retention during pediatric anesthesia in association with elevated plasma concentration of renin but not arginine–vasopressin. The mechanisms are further explored in conscious and anesthetized ewes where we show that RSNA is increased by sevoflurane compared with when conscious. This is accompanied by renal sodium and water retention and decreased renal blood flow (RBF). Finally, we demonstrate that renal denervation normalizes renal excretory function and improves RBF during sevoflurane anesthesia in sheep. Taken together, this study describes a novel role of the renal sympathetic nerves in regulating renal function and blood flow during sevoflurane anesthesia.

## Graphical Abstract

**Graphical Abstract:**
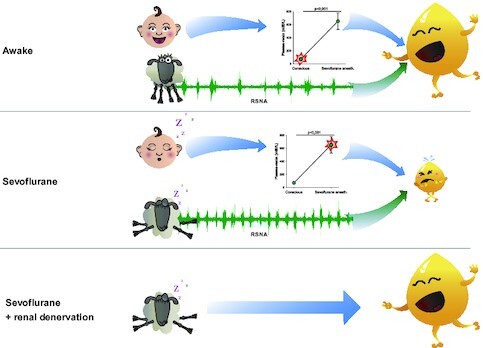

## Introduction

Acute kidney injury is a frequent complication of anesthesia and surgery linked to elevated risk of death and development of chronic kidney disease.^1^ Reduced urine output (less than < 0.5 mL/kg/h) for an extended period is generally an important indicator of AKI.^2^ Oliguria is also often interpreted as a sign of hypovolemia that in clinical practice warrants intravascular fluid resuscitation.^3^ Because anesthesia is known to reduce diuresis even in patients with normal kidney function and hydration status, urine output may be of limited value in detecting AKI during surgery and the use of volume loading to correct it is often to no effect or may even be detrimental.^4–6^ In the group of patients having oliguria during surgery there appears to be a gradation between those who progress to severe postoperative AKI and those who recover swiftly without any persisting renal dysfunction.^5,7^ Thus, enhanced knowledge concerning the mechanisms regulating urine output during anesthesia and surgery is likely to translate into improved care of surgical patients.

Current concepts hold that urine output is decreased during surgery by anesthesia-induced hypotension, mechanical ventilation and the stress due to surgical trauma, with a common pathway being the release of arginine–vasopressin (AVP).^8,9^ However, it has been demonstrated that volatile anesthetics, rather than mechanical ventilation, promote fluid accumulation.^10^ It was also recently reported that anesthesia affects renal oxygenation and renal blood flow (RBF) without producing hypotension.^11^ Finally, because reduced urine output in relation to surgery is accompanied by impaired renal sodium excretion ^12,13^ it is unlikely that AVP, that mainly regulates water but not sodium reabsorption, is the major mediator of the impairment in renal excretory function during anesthesia.

Depression of the autonomic nervous system is a global effect of anesthesia and can result in systemic vasodilation.^14,15^ Paradoxically, the volatile anesthetic isoflurane has been shown to increase renal sympathetic nerve activity (RSNA) while both cardiac and lumbar sympathetic nerve activity are decreased ^16^ (and unpublished observations). The potential result of increased RSNA would be augmented renal tubular water- and sodium-reabsorption and renal vasoconstriction, much like what is observed during surgery. Thus, we hypothesized that AVP-release is not the only factor mediating antidiuresis during volatile anesthesia. Instead, or in addition, elevated RSNA may be an important mechanism for renal water and sodium retention induced by sevoflurane.

Here, we explore the influence of one of the most used anesthetic agents, sevoflurane, on regulation of RSNA and the putative functional consequences on renal function and fluid balance. Initially, we confirmed diminished urinary output and sodium excretion and elevated levels of renin in children undergoing hypospadias surgery under sevoflurane anesthesia. Then, the investigation was expanded to invasive measurements of RSNA, renal excretory function, RBF, and cardiovascular function in conscious and anesthetized sheep. In sevoflurane anesthetized sheep, increases in RSNA were accompanied by decreases in urinary water and sodium excretion. To further analyze the decreased renal excretory response, the kidneys were denervated and agents selectively antagonizing both circulating AVP and angiotensin II, respectively, were used.

## Materials and Methods

This paper contains investigations in three settings. First, a human clinical investigation with eight children undergoing hypospadias surgery. The second, a cross-over study with repeated experiments in seven chronically instrumented ewes with intact renal nerves. Third, an additional seven sheep with similar instrumentation but with the addition of bilateral renal denervation.

## Water and Sodium Regulation in Pediatric Hypospadias Surgery

### Study Subjects

A total of eight male children > 10 kg,  <50 kg, and age of >1, <12 yr of age, with no prior illnesses (American Association of Anesthesiology class < 2), undergoing hypospadias surgery were enrolled in the study following informed consent signed by their legal guardians. Exclusion criteria were hypersensitivity/allergy to the anesthetics included in the study, insufficient knowledge of the Swedish language or inability of the parents to assimilate information about the study. This group of patients was chosen because of its relatively homogenous composition. We included previously healthy pediatric patients subjected to minor surgery and general anesthesia but with an anticipated modest inflammatory response to the procedure. In our clinical practice we have also previously noted a diminished urinary output in pediatric surgery under general anesthesia. The study was approved in advance by the regional ethics committee in Uppsala, Sweden. Ref 2015/225/1 and registered at clinicaltrials.gov, ID NCT02571426. The Declaration of Helsinki and its subsequent revisions were followed. STROBE (The Strengthening the Reporting of Observational Studies in Epidemiology) guidelines were followed for reporting.

### Experimental Protocol

**Figure 1.**
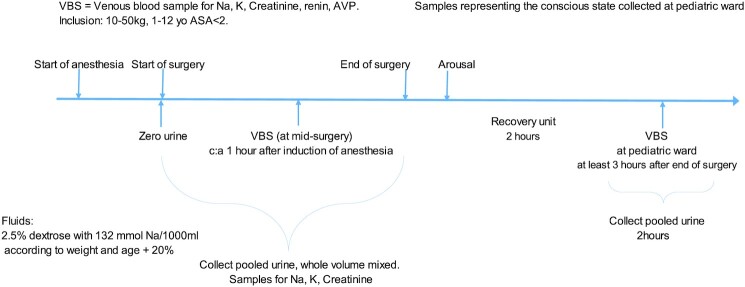
Schematic description of the study of the influence of volatile anesthesia on urine output and handling of sodium in pediatric elective outpatient hypospadias surgery. After initiation of sevoflurane anesthesia the urine bladder was emptied with a remaining Koyle stent. A venous blood sample was collected half-way into surgery (anticipated) and later analyzed for Na^+^, K^+^, creatinine, renin, and arginine–vasopressin (AVP). Throughout the anesthesia-period urine was collected for determination of urine output and analyses of Na^+^, K ^+^, and creatinine. After surgery the anesthesia was discontinued and the children were awakened in the operating room. They were then transferred to the recovery unit where they stayed for at least 2 h before they were again transferred to the pediatric ward. At the pediatric ward urine was collected for 2 h and venous blood was sampled 1 h after urine sampling was commenced.

The study was performed without disrupting the routine surgery. Anesthesia was induced with intravenous injection of propofol (Propofol–Lipuro 20 mg/mL,  B. Braun,  Melsungen,  Germany; 2–3 mg/kg) or when appropriate sevoflurane (Sevorane, AbbVie Inc., North Chicago, IL) administered through a breathing mask. All children were routinely given maintenance anesthesia with sevoflurane and caudal block with bupivacaine 1.25–2 mg/kg (Marcain 2.5 mg/mL,  Aspen Nordic, Ballerup, Denmark). Minimal Alveolar Concentration (MAC) of sevoflurane was measured continuously and is reported as the mean value during anesthesia. Tidal volume was set at start of anesthesia and not changed. Heart rate was measured via electrocardiogram and is reported as beats per minute (bpm). Systolic blood pressure was measured by the arm-cuff method and is reported as the mean value during surgery in mmHg. The amount of blood loss was estimated at the end of surgery. Under anesthesia, patients were equipped with a midline venous catheter for the collection of blood samples, and as part of the surgical protocol, a Koyle stent which was used for collection of urinary samples and measurement of urinary output. Urine was collected continuously during surgery and during a 2 h period in the pediatric ward where the patients were transferred postsurgery after at least 2 h in the recovery unit. A pooled urine sample was obtained for each period. Blood was sampled at mid-surgery and after 2 h of urine sampling in the pediatric ward (ie, at least 3 h after awakening from anesthesia). Blood samples were analyzed for Na^+^, K^+^, osmolality, renin and arginine-vasopressin. All patients received a standardized fluid regimen of buffered dextrose 25 mg/mL,  sodium 132 mmol/L,  at a fluid rate calculated by weight and age based on basal needs according to Holliday–Segar,^17^ with the addition of 20% during the entire study protocol (for example, a 12 kg child would get 45.8 mL/h according to Holliday–Segar, and in our protocol an additional 20%. In total 55 mL/h which corresponds to 4.6 mL/kg/h) Figure 1.

## RSNA, RBF, and Renal Excretory Function in Conscious and Anesthetized Ewes

Next, we conducted a set of experimensts in conscious and sevoflurane anesthetized surgically prepared sheep to further explore the mechanism of the observed water and sodium retention in patients during anesthesia and surgery. Understanding the acute physiological role of anesthesia may be challenging because it requires investigations in both conscious and anesthetized individuals. The advantage of using sheep is that they are medium-sized animals that allows for chronic surgical preparation without interference with normal physiology. Properly handled sheep can be studied both when conscious and un-stressed as well as during anesthesia, serving as their own controls.

### Study Subjects

A total of seven adult Gotland ewes (35–66 kg body weight) were housed in individual metabolism cages in association with other sheep. Experiments were initiated when the sheep were accustomed to laboratory conditions and human contact after at least a month. Sheep were fed a diet of oaten chaff (800 g/d) and hay, with the addition of 7g NaCl/d and water *ad libitum*. The experiments were approved in advance by the regional ethics committee in Stockholm, Sweden and adhere to the European Union directive 86/609/EEG and the European Council convention ETS 123. Ref N362/09. The ARRIVE guidelines (Animal Research: Reporting of In Vivo Experiments) were followed for reporting. All sheep underwent both conscious and anesthetized experiments with each sheep serving as its own control. They were randomized to start with either conscious or anesthetized experiments (Randomizer wheel, iPhone application by Rodskagg).

### Surgical Preparation

Prior to the study all sheep primarily underwent surgical preparation with a carotid arterial loop by externalizing the carotid artery in a fold of skin for facilitated access to arterial blood samples and monitoring of arterial blood pressure. Further surgical preparation included instrumentation for renal nerve recordings, measurement of RBF, and a catheter for renal venous blood sampling. For measurement of renal arterial blood flow, a perivascular flow probe (PS-series, according to vessel size, Transonic Systems Inc., Ithaca, NY) was implanted around the left renal artery. The two surgical procedures were separated by at least 1 wk. Prior to surgery they were premedicated with acepromazine (Plegicil Vet, Pharmaxim AB, Helsingborg, Sweden) 10 mg/mL, 0.4–0.6 mL/kg, and further analgesia with fentanyl 200 µg before the start of surgery, and repeated if necessary. For all surgery on the sheep, anesthesia was induced with an intravenous injection of sodium thiopental (Pentothal Natrium, Abbott, Lake County, IL) 500–1500 mg, and following endotracheal-intubation, the anesthesia was maintained with isoflurane (Isoflurane Baxter, Baxter International Inc., Deerfield, IL) 1.5%–2% isoflurane/O2. For the renal nerve recordings, the left renal artery was exposed via a paracostal retroperitoneal approach under general anesthesia. Recording electrodes and a metal ground plate were placed and fixed as previously described.^18^ In all operations, the sheep were treated with intramuscular antibiotics dihydrostreptomycin and penicillin G procaine (Streptocillin Vet 250 mg/mL + 200 mg/mL, Boehringer Ingelheim Animal Health Nordics A/S, Copenhagen, Denmark), 0.1 mL/kg at the start of surgery and for 2 d postoperatively. Postsurgical analgesia was maintained with intramuscular injections of buprenorphine (Temgesic 0.3 mg/mL,  Indivior Inc.,  North Chesterfield,  VA) 0.005–0.01 mg/kg 30 min prior to end of anesthesia and every 4–8 h the first 12 h, and then as needed until pain free. Additionally, carprofen (Rimadyl Bovis vet 50 mg/mL, Zoetis Finland Oy, Helsinki, Finland) 0.7–4 mg/kg was administered once daily for 3 d. Each time prior to the start of the study protocol, the sheep were equipped with an intravenous catheter in the left jugular vein, an arterial catheter in the externalized carotid artery, a pulmonary artery-catheter via the right jugular vein, and a Foley catheter in the bladder as previously described.^19^

### Experimental Protocol

After cannulation the sheep were allowed an hour for stabilization and equilibration. Anesthetic experiments were carried out under supervision of anesthetic depth and monitoring of stable circulatory conditions and lack of blinking, lacrimal reflexes or movement. Conscious sheep had water *ad libitum* and anesthetized sheep received basal fluid replacement with Ringer's acetate solution at 5 mL/kg/h. After initial baseline recording of RSNA, central venous pressure (CVP), pulmonary capillary wedge pressure, pulsatile arterial pressure, mean arterial pressure (MAP), pulmonary artery pressure, heart rate, and blood gases (arterial, mixed venous, and renal venous blood), were collected. Anesthetized sheep were connected to a Maquet Flow-I anesthesia machine (Getinge AB, Gothenburg, Sweden). Respiratory settings included tidal volume 7 mL/kg, Positive End Expiratory Pressure (PEEP) 5 cm H_2_O, with inspired oxygen concentration and breathing frequency adjusted to achieve normal arterial blood gas values. Measurements of tidal volume, breathing frequency, inspiratory oxygen concentrations, and CO_2_ levels were noted in the study protocol. Incremental doses of phenylephrine were administered intravenously to increase arterial pressure while measuring the RSNA response until the signal reached its minimal value. Recordings were made at baseline but also after a crystalloid fluid load (Ringer's Acetate 20 mL/kg was administered over 30 min). The rationale for the volume expansion was dual. First we wanted to assure that the anticipated oliguria was not due to appropriate afferent reflex input secondary to hypovolemia. Secondly, because a common clinical intervention for oliguria is fluid resuscitation we set out to investigate if the effects of sevoflurane on RSNA and renal function could be influenced by fluid management.

**Figure 2.**
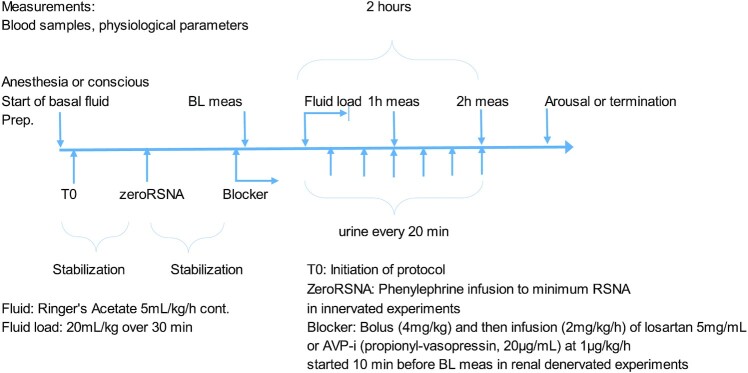
Schematic description of the experimental protocol for the experiments in sheep. The same study protocol was used in anesthetized and conscious sheep with or without renal denervation. After a stabilization period phenylephrine was infused to obtain minimum RSNA level (ZeroRSNA) in order to discriminate background noise from actual signal burst (this was not done in the renal denervation experiments). After baseline measurements (BL meas) Ringer's acetate solution (20 mL/kg) was infused over 30 min. Urine was collected every 20 min for determination of urine output and analyses. Measurement of physiological parameters and blood sampling was performed 1 and 2 h after start of infusion. At the end of the protocol anesthesia was discontinued (if sevoflurane experiments were performed) and the sheep were allowed back in their cages. Blocker = losartan potassium (5 mg/mL) at a rate of 2 mg/kg/h after a bolus of 4 mg/kg (over 10 min) or Propionyl-D-Tyr(Et)-Phe-Val-Asn-Abu-Pro-Arg-Arg-NH₂ (20 µg/mL) at a rate of 1 µg/kg/h was administered in some of the experiments on renal denervated sheep.

Measurements were repeated at 1 and 2 h after start of fluid administration. Urine was collected at baseline, after 1 h and then in 20-minute intervals for 2 h after start of fluid load. RSNA was recorded for 10 min periods at baseline and every 30 min after start of fluid load. This was done until 2 h after start of fluid load. At the end of the last experiment animals were deeply anaesthetized with sodium thiopental and sacrificed with an overdose of potassium chloride Figure 2.

### Recording of RSNA

RSNA was recorded as previously described.^18^ Sympathetic nerve activity (5000 Hz), arterial blood pressure (250 Hz), and RBF (250 Hz) were digitally sampled on a computer using a CED Micro 1401 interface and Spike 2 software (Cambridge Electronic Design, Cambridge, UK).

In three anesthetized sheep, recordings were made during both mechanical ventilation and spontaneous breathing in regard to effects on RSNA. Examples of those recordings are shown in **Figure S1**A–C.

## RBF and Renal Excretory Function in Conscious and Anesthetized Ewes After Bilateral Renal Denervation

In a third investigation, we wanted to mechanistically explore the hypothesis that RSNA mediates the renal excretory effects of sevoflurane. Sheep were subjected to renal denervation and treated with intravenous antagonists of vasopressin or angiotensin II. For comparison, we investigated the response to the same fluid load (20 mL/kg Ringer's acetate solution for 30 min) after 2 h.

### Study Subjects

An additional set of seven adult Gotland ewes (51–67 kg body weight) were kept under the same conditions as described in 2A. The experiments were approved in advance by the regional animal ethics committee in Uppsala, Sweden. Ref C148/15.

### Surgical Procedures

All sheep were subjected to the same procedures as described in 2B above, except for the renal nerve electrode preparation. Instead, the kidneys were denervated by cutting all the visible renal nerves bilaterally, leaving a gap of at least 2 cm. This was followed by application of 10% phenol in 85% ethanol in 0.9% NaCl around the full circumference of the renal artery and the cut nerve endings. Renal denervation was confirmed post mortem by absence of tyrosine hydroxylase as determined by immunohistochemistry.

### Experimental Protocol

All sheep underwent four experimental protocols that is conscious, anesthetized, anesthetized + angiotensin II receptor blocker (ARB), and anesthetized + arginine-vasopressin inhibitor (AVP-i). The ARB used was losartan potassium (Merck KGaA, Darmstadt, Germany) diluted to 5 mg/mL in 0.9% NaCl and infused intravenously at a rate of 2 mg/kg/h throughout the experiment after a bolus of 4 mg/kg. For antagonism of the vasopressin V2-receptor Propionyl-D-Tyr(Et)-Phe-Val-Asn-Abu-Pro-Arg-Arg-NH₂ (20 µg/mL in 0.9% NaCl, Bachem AG, Bubendorf, Switzerland) was infused intravenously at a rate of 1 µg/kg/h throughout the experiment. Doses of both compounds were based on previous studies in sheep.^20,21^ Each sheep served as its own control. Sheep were randomized to the order in which the experiments were performed and there was a minimum of 24 h between experiments. The experiments were carried out identical to the description in 2C above with the exception of RSNA-measurements. At the end of the last experiment animals were deeply anaesthetized with sodium thiopental and sacrificed with an overdose of potassium chloride Figure 2.

Immediately postmortem, renal tissue samples were collected and frozen at −80°C. Immunohistochemistry of the renal tissue was performed to verify renal denervation. Frozen kidney samples (−80°C) were thawed in 4% formalin for approximately 24 h during which formalin was changed once. The samples were then dehydrated, and paraffin embedded. Briefly, the tissue pieces were sectioned at 4 µm thickness, mounted onto slides, deparaffinized, rehydrated, and washed. The slides were pretreated in sodium-citrate buffer (pH 6) at 92°C for 20 min, then left to cool for 20 min at room temperature after which they were washed in distilled water. Endogenous peroxidase activity was extinguished with 3% hydrogen peroxide in PBS (0.01 mol/L phosphate, 0.15 mol/L NaCl, pH 7.4) 5 min at room temperature in the dark. After washing with deionized water and PBS, the sections were then incubated with polyclonal antibodies directed against tyrosine hydroxylase (AB152, Merck, Solna, Sweden) diluted in PBS-Tween (pH 7.6), overnight at 4°C. As negative controls, the primary antibody was substituted with nonimmune rabbit serum (X0936, DAKO Sweden AB, Stockholm, Sweden). After rinsing in PBS, visualization of antibody-antigen complexes was done with the Dako EnVision Detection System (K5007, DAKO Sweden AB). Using the kit reagents, the sections were incubated with horseradish peroxidase-conjugated secondary antibodies for 30 min at room temperature, and afterwards treated with color developer 3,3-diaminobenzidine before the sections where counterstained with Mayer´s hematoxylin. The sections were evaluated using a Nikon Eclipse Ci-L microscope (Bergman Labora AB Stockholm, Sweden) and the staining intensity subjectively graded. The veterinary pathologist doing the immunohistochemical evaluation had no prior knowledge about the treatment of individual animals.

## Blood and Urine Analysis

Venous blood samples in all studies (human and sheep) were immediately placed into prechilled tubes and centrifuged at 2400 g for 10 min at 4°C (Eppendorf centrifuge 5702, Hamburg, Germany). Plasma was then frozen and stored for later analysis. Urine osmolality was directly measured in an osmometer (Auto & Stat Om 6010 osmometer; Kagaku Co., Kyoto, Japan). Creatinine (enzymatic method) and sodium and potassium (indirect ion-selective method) were analyzed in a certified chemical laboratory at Akademiska University Hospital, Uppsala, Sweden in Architect c8000 and c16000 instruments (Abbott, Abbott Park, IL)

Urine samples for all studies were frozen and stored for later analysis. Urine osmolality, creatinine and electrolytes were determined as above.

Plasma Arg^8^-Vasopressin was analyzed by competitive ELISA according to the manufacturer's recommendations (ADI-900–017, Enzo Life Sciences, Farmingdale, New York, NY). The total coefficient of variation (CV) of the assay was approximately 7%. Plasma renin concentration was analyzed on Liaison XL (DiaSorin, Saluggia, Italy) with reagents from the same manufacturer. The total CV of the assay was 2% at 26 mU/L. All assays were performed in a blinded manner without knowledge of clinical data.

### Data Analysis

RSNA was analyzed in MATLAB software (Mathworks, Natick, MA). Samples (>5-minute-long) of the explored points of measurement (BL, 30, 60, 90, and 120 min) were imported to MATLAB, where outliers were removed. Linear detrending was then performed on the large data sets to account for downward drift over time. After full-wave rectification of the RSNA signal a moving average over 20 ms was calculated and mean moving average values for each recording period was determined. The unique zeroRSNA for each experiment was subtracted from each of the meaned moving averages for the periods of measurement. RSNA activity was then expressed as % of baseline activity in the periods following the fluid load. The smallest burst was identified in the entire recording from a spreadsheet of data, and its correct position in the cardiac cycle and absence of artifacts were confirmed visually. The rectified and integrated area between the diastolic pressures bracketing this burst was noted, and this area was taken as the minimum area for the definition of a burst. When the rectified and integrated area between any heartbeats was greater than the minimum area, this was determined to constitute a burst. The burst incidence was calculated as the number of bursts per 100 heart beats. The burst frequency was calculated as the number of bursts per minute. As previously described, burst amplitude was calculated as the integrated area under the curve for each burst.^22^ The largest burst during baseline recordings for each sheep was used as reference and the sizes of all remaining bursts were calculated as a percentage of this burst. The relative burst amplitudes were calculated by determining the frequency distribution of all the bursts over 5 min during the baseline period, at 30 min and at 120 min after commencing fluid loading with Ringer's Acetate.

Free water clearance, (C_H2O_) was calculated as: urinary flow in mL/min—((U-osm/P-osm) * urinary flow in mL/min). FENa calculated as (P-crea * U-Na)/(P-Na * U-crea), all in mmol/L.

Creatinine clearance was calculated as urinary flow in mL/min * U-crea/P-crea, and then normalized according to body surface area, which for sheep was calculated according to Berman as 0.09 x W^0.67^.^23^ Renal vascular resistance was calculated as (MAP-CVP)/RBF and total peripheral resistance was calculated as (MAP-CVP)/CO.

### Statistical Analysis

All statistical calculations were made in Statistica version 13.5 (Statsoft Europe, Hamburg, Germany). Data are presented as mean ± standard deviation (descriptive tables and text) or mean ± standard error of the mean (figures) after it was controlled for normality by visual inspection of a histogram plot of absolute values and a quantile versus quantile plot generated for each parameter. The significance level was set as *P* < .05. All children and sheep were examined both conscious and under sevoflurane anesthesia. Within-subject pairwise comparisons between the conscious and anesthetized were performed using Student's paired *t*-test. Changes in parameters over time in sheep with intact nerves (set 2 of the experiments described above) were analyzed according to a two-way repeated-measures ANOVA, the repeating variables being time and treatment (anesthesia/conscious). Changes over time were interpreted as an effect of the fluid bolus and differences in treatment as an effect of sevoflurane anesthesia. A significant interaction between time and treatment was interpreted as an effect of sevoflurane on the response to fluid loading. If the overall F ratio was significant, Tukey's honestly significant difference (HSD) posthoc test was used for comparisons of means. In case of significant interaction between time and treatment, simple main effects were examined to investigate differences between anesthesia and conscious at baseline and after fluid loading. As this procedure consists of multiple testing, the *P*-values were adjusted according to Bonferroni. When comparing renal denervated sheep (with or without the AVP- or AngII-antagonists, set 3 of the experiments described above) with sheep with intact renal nerves a one-way ANOVA followed by Tukey's HSD posthoc test was used to analyze absolute differences in parameters after volume loading but also differences in the effect of volume loading. The sheep were chronically instrumented, and over time some measurements were inevitably lost. In the denervation experiments, the ultrasonic flow probes malfunctioned in three out of seven sheep in later experiments. This lead to lost data from one sheep in the conscious + renal denervation (RDN) and sevoflurane + RDN experiments and lost data from three sheep in the sevoflurane + RDN + los and sevoflurane + RDN + AVP-i experiments. This data was treated as missing.

## Results

### Water and Sodium Regulation in Pediatric Hypospadias Surgery

**Figure 3.**
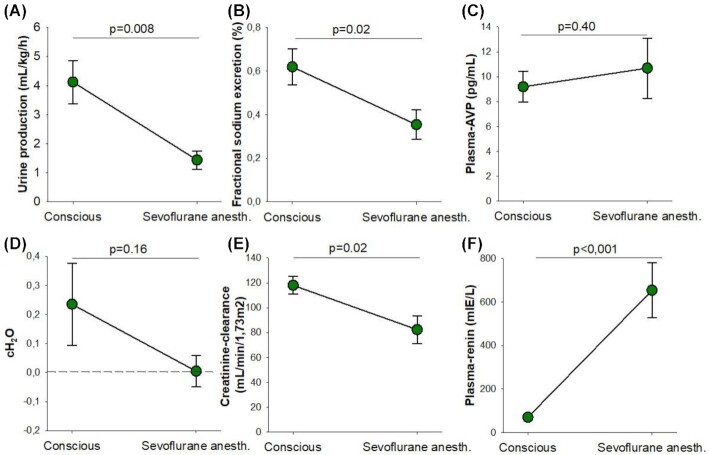
Renal excretory function and plasma levels of arginine–vasopressin and renin in conscious and sevoflurane anesthetized children Urine output (mL/kg/h) (A), fractional sodium excretion (%) (B), plasma levels of arginine–vasopressin (pg/mL) (C), free water clearance (mL/min) (D), creatinine clearance (mL/min/1.73 m2) (E) and plasma levels of renin (mIE/L) (F) in children, conscious and under sevoflurane anesthesia (*n* = 8 with repeated measurements conscious and under sevoflurane anesthesia in the same individual). Data are expressed as mean ± standard error of the mean with the significance level set as *P* < .05. Within-subject pairwise comparisons between the conscious and anesthetized state were performed using Student's paired *t*-test. Creatinine clearance was calculated as urinary flow in mL/min * U-crea/P-crea, and then normalized according to body surface area. CH2O was calculated as urinary flow in mL/min—((U-osm/P-osm) * urinary flow in mL/min). FENa was calculated as (P-crea * U-Na)/(P-Na * U-crea), all in mmol/L.

All children underwent the surgical procedure without any adverse or unanticipated events. Compared with the conscious state, surgery and sevoflurane anesthesia markedly reduced urinary output (by 65 ± 22%; mean ± standard deviation; Figure 3A), fractional sodium excretion (by 39 ± 29%, Figure 3B) and absolute sodium excretion (by 49 ± 43%, 0.15 ± 0.05 mmol/kg/h to 0.07 ± 0.07 mmol/kg/h, *P* = .03). This was associated with a pronounced increase in p-renin concentration (+1017 ± 681%, Figure 3F) but no significant change in p-AVP (Figure 3C). Free Water Clearance (C_H2O_, Figure 3D), was not significantly affected by anesthesia and surgery which corroborates the AVP-finding. In the absence of changes in arterial pressure, the two dominating stimuli for renin release are RSNA and decreased glomerular filtration rate (GFR), the latter via the macula densa mechanism.^24^ Since creatinine clearance was reduced compared with the conscious state (−30 ± 25%, Figure 3E) and RSNA was not measured, this set of investigations could not discriminate between increased RSNA and decreased GFR as the cause of the increase in p-renin. Adequate intravascular fluid management was performed to keep blood pressure and heart rate within adequate limits for the age of the patients (Table 1).^25^ Thus, there was no apparent indication of change in afferent input from baroreceptors or volume receptors that could stimulate RSNA.

**Table 1.** Demographic Characteristics and Per-operative Parameters of Eight Male Pediatric Patients Subjected to Hypospadias Surgery Under Sevoflurane Anesthesia

	Mean	SD
Age (months)	33.6	27.8
Duration of anesthesia (min)	145	18
Blood loss (mL)	9.4	2.0
MAC	1.1	0.1
Tidal volume (mL/kg)	6.9	0.6
Heart rate (bpm)	120	13
Systolic blood pressure (mmHg)	80	11

Values are represented as means and standard deviation (SD). Minimal Alveolar Concentration (MAC) of sevoflurane was measured continuously and is reported as the mean value during anesthesia. Tidal volume was set at start of anesthesia and not changed. Heart rate was measured via electrocardiogram and is reported as beats per minute (bpm). Systolic blood pressure was measured by the arm-cuff method and is reported as the mean value during surgery in mmHg. The amount of blood loss was estimated at the end of surgery.

### RSNA, RBF, and Urine Output in Conscious and Anesthetized Ewes

**Figure 4.**
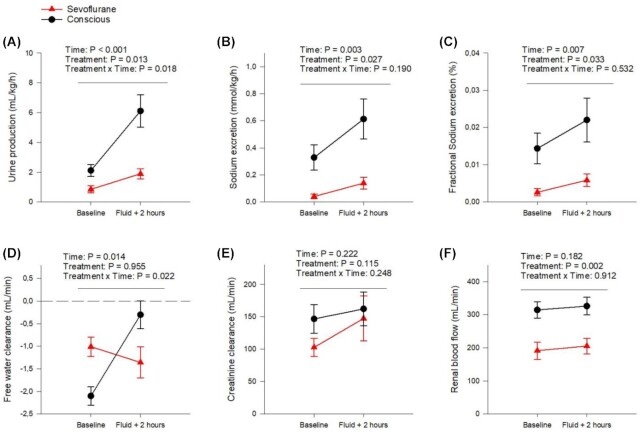
Renal excretory function and blood flow in conscious and sevoflurane anesthetized sheep. Urine output (mL/kg/h) (A), sodium excretion (mmol/kg/h) (B), fractional sodium excretion (%) (C), free water clearance (mL/min) (D), body surface area adjusted creatinine clearance (mL/min) (E) and RBF (mL/min) (F) in conscious sheep (filled black connected circles) compared with the same sheep under sevoflurane anesthesia ((filled red connected triangles). Comparisons were made at baseline and 2 h after an intravenous fluid load with 20 mL/kg Ringer's acetate solution infused over 30 min. Data are presented as mean ± SEM with the significance level set as *P* < .05. Changes in parameters over time were analyzed according to a two-way repeated-measures ANOVA, the repeating variables being time and treatment (anesthesia/conscious). Changes over time were interpreted as an effect of the fluid bolus and differences in treatment as an effect of sevoflurane anesthesia. A significant interaction between time and treatment was interpreted as an effect of sevoflurane anesthesia on the response to fluid loading. Creatinine clearance was calculated as urinary flow in mL/min * U-crea/P-crea, and then normalized according to body surface area. CH2O was calculated as urinary flow in mL/min—((U-osm/P-osm) * urinary flow in mL/min). FENa was calculated as (P-crea * U-Na)/(P-Na * U-crea), all in mmol/L. Renal vascular resistance was calculated as (MAP-CVP)/RBF. *N* = 7.

During experiments on conscious animals, the ewes stood in their cages and showed no signs of discomfort. Similar to the children the sheep had a markedly reduced urinary output when anesthetized with sevoflurane, compared with conscious (−52 ± 29% at baseline, *P* = .03, Figure 4A). Urine output also increased more after the fluid load when conscious compared with during anesthesia (by 3.99 ± 2.02 mL/kg/h for conscious and by 1.04 ± 0.88 mL/kg/h when anesthetized, Figure 4A). Sevoflurane anesthesia reduced both absolute sodium excretion (by 85 ± 12%, Figure 4B), and fractional sodium excretion (by 78 ± 16%, Figure 4C). Further, both absolute and fractional sodium excretion were increased in response to the fluid load with no intergroup difference (in absolute sodium excretion the increase was 0.26 ± 0.17 mmol/kg/h for conscious and 0.10 ± 0.10 mmol/kg/h with sevoflurane anesthesia, and in FENa the increase was 0.007 ± 0.007 FENa% for conscious and 0.003 ± 0.005 FENa% with sevoflurane anesthesia, Figure 4B and C). Thus, it appears that sevoflurane reduces both water and sodium excretion and that the expected diuretic response to fluid loading was blunted by this anesthetic. At baseline C_H2O_ was negative with and without anesthesia, indicating urinary concentration. Interestingly, conscious sheep had lower C_H2O_ as compared with when anesthetized with sevoflurane (50 ± 27%, *P* = .008, Figure 4D). The response to the fluid load when the sheep were conscious was, as expected, an elevation in C_H2O_ (by 1.80 ± 1.23 mL/min, Figure 4D). However, this effect was different from when the sheep were anesthetized since fluid loading did not significantly alter C_H2O_ under sevoflurane anesthesia (Figure 4D). There were no significant differences in creatinine clearance between the treatments (Figure 4E). Nor was there any difference detected between values at baseline. The intra-group variations were larger compared with the same measurement in the children.

As reported previously, RBF was decreased with volatile anesthesia compared with when conscious.^16,26^ This effect was also observed in the current experiments where RBF was reduced by 38 ± 19% at baseline (*P* =.004, Figure 4F). Our results further demonstrate that this effect is not due to hypovolemia since a major fluid load which increased pulmonary capillary wedge pressure (PCWP) with no intergroup difference (by 36 ± 52%, *P* = .003, Table S1) did not significantly change RBF in either the conscious or the anesthetized state.

**Figure 5.**
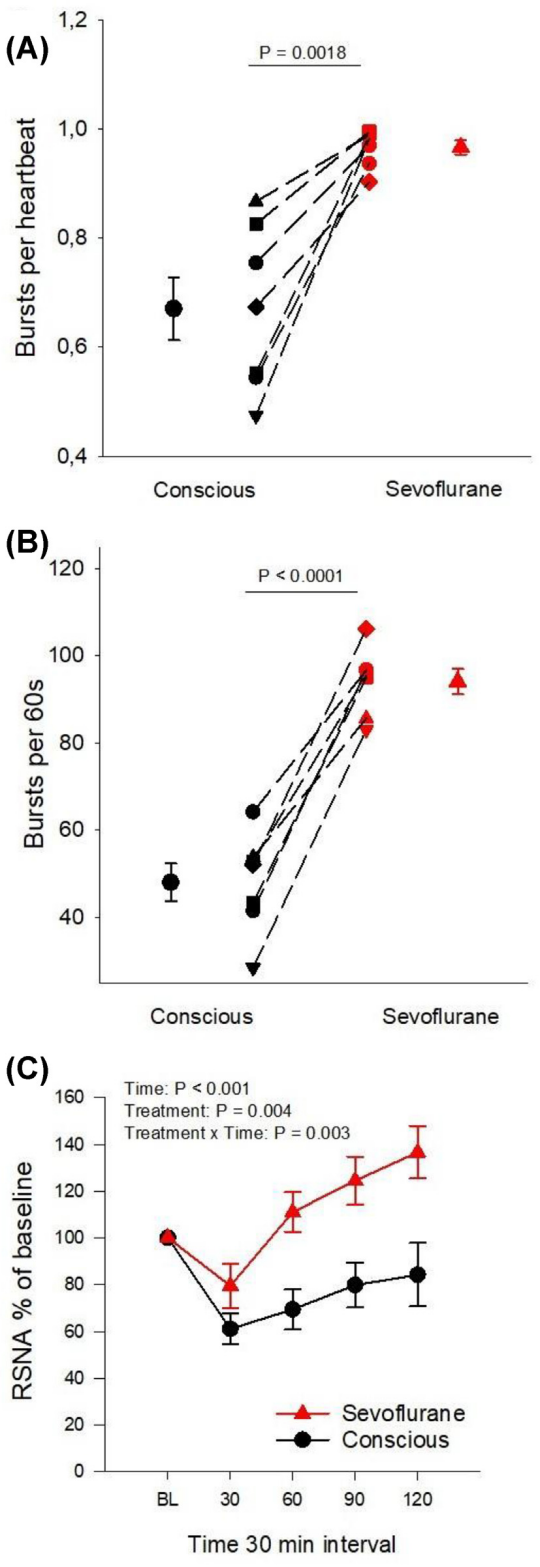
Comparative RSNA analysis in conscious and sevoflurane anesthetized sheep (A) Burst count per heartbeat at baseline, (B) Burst count per minute at baseline, and (C) change in integrated RSNA voltage as percentage from baseline after fluid load of Ringer's acetate solution (20 mL/kg over 30 min) in sevoflurane anesthetized (filled red symbols) and conscious (filled black symbols) sheep. Dashed lines in (A) and (B) connect symbols for individual sheep in the different experiments. Mean values are displayed with bars for standard error of the mean. Differences between the conscious state and sevoflurane anesthesia were analyzed with within-subject pairwise comparisons using Student's paired *t*-test (*P*-value in figures A and B). Changes in parameters over time in C) were analyzed according to a two-way repeated-measures ANOVA using time-points 30 and 120, the repeating variables being time and treatment (anesthesia/conscious). Changes over time were interpreted as an effect of the fluid bolus and differences in treatment as an effect of sevoflurane anesthesia. A significant interaction between time and treatment was interpreted as an effect of sevoflurane anesthesia on the RSNA-response to fluid loading (Treatment x Time: *P*-value in C). The significance level was set as *P* < .05.

RSNA was significantly higher with sevoflurane anesthesia compared with when conscious by three modalities: burst frequency (+105 ± 48%), burst incidence (+51 ± 35%) and finally percentage change from baseline of RSNA (moving average voltage) after fluid load (Figure 5A–C). Burst frequency provides a measurement of overall RSNA. However, sympathetic bursts are associated with heart rate. The bursts rarely occur more than once during each heart cycle but overall RSNA may be affected by changes in heart rate. By analyzing bursts per heartbeat (burst incidence) any effect of heart rate on differences in RSNA between groups can be excluded. In many anesthetized sheep every heartbeat resulted in a burst (1:1 ratio), evoking maximum sympathetic stimulation similar to severe hypotension or hypovolemia (Figure 5A and **Figure S1**). In addition, although RSNA was initially decreased by the fluid load both with sevoflurane anesthesia (to 79 ± 25% of baseline) and without (to 61.07 ± 17.06% of baseline) the reduction was blunted by sevoflurane (Figure 5C). A total of 2 h after the intravenous infusion was started, RSNA had increased more during anesthesia compared with the conscious state (to 136.61 ± 29.87% versus 84.25 ± 35.89% of baseline at 120 min after fluid load, *P* = .006). This indicates a tonic stimulation of RSNA by sevoflurane that can be reduced but not normalized by afferent input from volume- and baro-receptors. Of note is that sevoflurane anesthesia compared with the conscious state shifted the frequency distribution of burst amplitudes to higher levels at baseline (23.90 ± 9.47 versus 8.67 ± 10.44% of total burst number were 80% of maximum burst amplitude, *P* = .005, **Figure S2**). This suggests that besides a high burst frequency, an increase in burst amplitude may have contributed to overall increase in RSNA during sevoflurane-anesthesia.

The different effects of volatile anesthesia and mechanical ventilation on urinary excretion have been investigated previously.^10^ However, to assure that the increased RSNA was not due to mechanical ventilation, sympathetic activity was recorded during anesthesia at the same end-tidal concentration of sevoflurane in three spontaneously breathing anesthetized sheep. RSNA was equally increased without mechanical ventilation (original recordings in S-Figure 1).

Hemodynamic data are shown in Table S1. Cardiac output (CO) was lower with sevoflurane than without anesthesia at baseline (−19 ± 14%, *P* = .02) and 2 h after fluid load (−13 ± 20%, *P* = .02). Mean arterial pressure (MAP), CVP, PA, or PCWP were not affected by sevoflurane and there were no interaction effects between time and treatment. Compared to the conscious state heart rate (HR) was higher during sevoflurane anesthesia at baseline (+25 ± 22%, *P* = .02) and 2 h after fluid load (+ 34 ± 24%, *P* = .008).

Total peripheral resistance (TPR) was increased during anesthesia compared with the conscious state at baseline (by 41 ± 40%, *P* = .02) but did not differ significantly 2 h after fluid load.

Renal vascular resistance (RVR) was increased by sevoflurane compared with when conscious at baseline (by 94 ± 58%, *P* = .007) and 2 h after fluid load (by + 65 ± 58%, *P* = .01).

Taken together the results of the investigations in the children and the sheep are consistent with a RSNA-stimulating effect of sevoflurane resulting in renin-release, renal vasoconstriction and sodium- and water-reabsorption without major effects on AVP-release.

### RBF and Renal Excretory Function in Conscious and Anesthetized Ewes After Bilateral Renal Denervation

**Figure 6.**
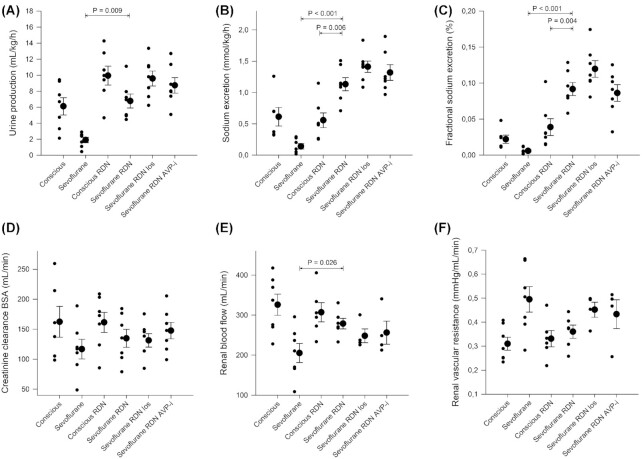
Renal excretory function and blood flow in conscious and sevoflurane anesthetized sheep with intact renal nerves and after renal denervation. Urine output (mL/kg/h) (A), sodium excretion (mmol/kg/h) (B), fractional excretion of sodium (%) (C), body surface area adjusted creatinine clearance (mL/min) (D), RBF (mL/min) (E) and renal vascular resistance (mmHg/mL/min) (F) investigated in conscious sheep with and without intact renal innervation, sevoflurane anesthetized sheep with and without renal innervation and renal denervated sheep with intravenous infusion of losartan (5 mg/mL in 0.9% NaCl,  4 mg/kg bolus followed by 2 mg/kg/h) or the vasopressin V2-receptor antagonist Propionyl-D-Tyr(Et)-Phe-Val-Asn-Abu-Pro-Arg-Arg-NH₂ (20 µg/mL in 0.9% NaCl, 1 µg/kg/h). Comparisons are performed 2 h after start of a fluid load (Ringer's Acetate, 20 mL/kg) to avoid any potential confounding effects of intravascular hypovolemia associated to anesthesia. Data are expressed as mean ± standard error of the mean (large filled circles with error bars) with the significance level set as *P* < .05. Values for individual sheep are illustrated by small filled circles. *N* = 7 in all except “conscious” in Sodium excretion, FENa and Creatinine clearance BSA where *N* = 6 due to a lost U-creatinine sample; in RBF and Renal vascular resistance where *N* = 6 in “conscious RDN” and “Sevoflurane RDN,” *N* = 4 in “Sevoflurane RDN los” and “Sevoflurane RDN AVP-i” due to probe failure. In RVR “conscious” two individual measurements are overlapping at 0.25 mmHg/mL/min and in creatinine clearance “sevoflurane RDN los” two individual measurements are overlapping at 113 mL/min. P values were derived from a one-way ANOVA followed by Tukey's HSD posthoc test if a significant main effect. Horizontal bars with *P*-values correspond to the two experiments linked by the ends of the bars. For sake of clarity only significant differences are indicated for the following comparisons: Sevoflurane versus Sevoflurane RDN, Sevoflurane RDN versus Sevoflurane RDN los, Sevoflurane RDN versus Sevoflurane RDN AVP-I; Sevoflurane RDN versus Conscious RDN and Conscious vs Conscious RDN. Creatinine clearance was calculated as urinary flow in mL/min * U-crea/P-crea, and then normalized according to body surface area. C_H2O_ was calculated as urinary flow in mL/min—((U-osm/P-osm) * urinary flow in mL/min). FENa was calculated as (P-crea * U-Na)/(P-Na * U-crea), all in mmol/L. Renal vascular resistance was calculated as (MAP-CVP)/RBF. RDN = renal denervation. Los = losartan (ARB). AVP-i = arginine-vasopressin-inhibitor (propionyl-vasopressin).

Urine output was significantly higher during anesthesia in sheep with denervated kidneys compared with sheep with innervated kidneys investigated in the first set of experiments both at baseline (from 0.86 ± 0.64 to 5.15 ± 4.50 mL/kg/h, *P* = .03) and after fluid load (from 1.90 ± 0.90 to 6.77 ± 2.31 mL/kg/h, Figure 6A). No additional significant effects on urine flow rate beyond denervation were found with ARB or AVP-i during anesthesia after volume loading (Figure 6A). There were no significant effects of denervation on the increase in urine output caused by volume loading in conscious or anesthetized sheep (Table S3). However, angiotensin II antagonism by losartan in denervated sheep increased urine output more in response to fluid loading compared with sheep with intact nerves (from 4.45 ± 1.17 to 9.58 ± 2.47 versus from 0.86 ± 0.64 to 1.90 ± 0.90 mL/kg/h, *P* = .03, Table S3).

Absolute and fractional sodium excretion during sevoflurane anesthesia were increased in renal denervated sheep compared with sevoflurane anesthetized sheep with intact innervation (Absolute: from 0.04 ± 0.04 to 0.68 ± 0.47 mmol/kg/h, *P* = .004 at baseline and from 0.14 ± 0.11 to 1.13 ± 0.28 mmol/kg/h after fluid and FENa%: from 0.003 ± 0.003 to 0.049 ± 0.028%, *P* = 0.001 at baseline and from 0.006 ± 0.004 to 0.092 ± 0.024% after fluid, Figure 6B and C). In addition, the increase in FENa in response to fluid loading was more pronounced in renal denervated sheep compared with renal innervated sheep under anesthesia (4.2 ± 2.2% versus 0.3 ± 0.5%, *P* = .03, Table S3). For absolute sodium excretion the difference in the response was not significant (*P* = .28, Table S3). Absolute and fractional sodium excretion were not significantly affected by renal denervation in conscious sheep (Figure 6B and C). Furthermore, there were no significant differences detected in the effect of intravenous Ringer's acetate solution on absolute sodium excretion or fractional sodium excretion in conscious sheep with or without renal innervation (Table S3). Taken together this indicates that RSNA contributes to decreased sodium excretion during sevoflurane anesthesia. There was no additional effect by adding ARB or AVP-i on absolute or fractional sodium excretion during anesthesia in renal denervated sheep (Figure 6B and C).

RBF was increased during anesthesia in sheep with renal denervation compared with sheep with innervated kidneys (by 49 ± 67%, Figure 6E). The reduction in RBF in sevoflurane anesthetized compared to conscious sheep was no longer significant after renal denervation (326 ± 70 mL/min vs 278 ± 32 mL/min, *P* = .16, Figure 6E). When conscious, no significant difference could be detected between sheep with RDN and innervated kidneys regarding RBF (Figure 6E). Adding intravenous ARB or AVP-i to renal denervated sheep under anesthesia did not cause further significant change in RBF (Figure 6E).

There was no difference detected in RVR between intact sheep and sheep with RDN during anesthesia (Figure 6F). Neither did we find a difference in RVR between conscious sheep with denervated kidneys and innervated kidneys (Figure 6F). For sheep with intact renal nerves, RVR was increased by 94% (± 58%, *P* = .007) when anesthetized compared with when the ewes were conscious. This increase was no longer detected after denervation (conscious RDN vs sevoflurane RDN; +13 ± 29%, *P* = .46, Figure 6F). Adding intravenous ARB or AVP-inhibition to renal denervated sheep under sevoflurane anesthesia did not cause further changes to RVR (Figure 6F). Nor were there any differences detected regarding the effect of fluid loading on RBF and RVR (Figure 6F). The fluid load had no significant effect on RBF or RVR in denervated sheep, with or without blockade of AngII or arginine-vasopressin.

Creatinine clearance (Figure 6D), and MAP (Table S2) were stable without significant differences between interventions or in the effect of volume loading. For further respiratory and circulatory variables, see Table S2.

### Immunohistochemistry

**Figure 7.**
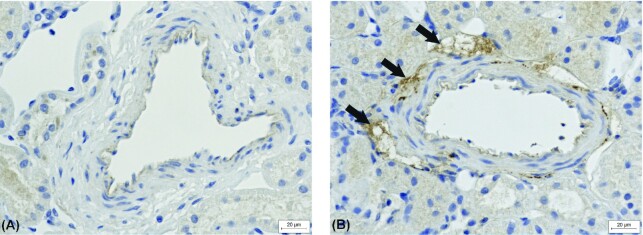
A typical example of immunohistochemistry staining against tyrosine hydroxylase in a periarterial area of kidney sections from (A) renal denervated sheep and (B) intact renal innervation (control). Area with positive staining indicated with arrows.

All denervated sheep (*n* = 7) lacked periarterial tyrosine hydroxylase but it was present in all control sheep, indicating successful denervation. A typical example of the tyrosine hydroxylase immunohistochemistry is shown in Figure 7.

## Discussion

Physiological mechanisms affected by modern anesthetics warrant further elucidation in order to improve surgical outcomes. This study explores the influence of a volatile anesthetic (sevoflurane) on RSNA, and the downstream impact on the renal regulation of sodium and water balance. The study was carried out by three complementary investigations in different settings. We provide evidence that sevoflurane substantially increases RSNA to near maximal levels (one burst for every heartbeat) and promotes release of renin. As a result, urinary sodium and water excretion are attenuated, which with unchanged creatinine clearance are likely due to increased tubular sodium and water reabsorption. The study in sevoflurane anesthetized pediatric patients describes reduced natriuresis in relation to increased p-renin concentration but unchanged p-AVP concentration. The findings of attenuated diuresis and natriuresis were experimentally repeated in sheep under sevoflurane anesthesia. It was demonstrated that reduced renal excretory function is associated with increased RSNA and decreased RBF. The effects on RSNA and RBF were not normalized by substantial crystalloid plasma volume expansion. In the last set of experiments renal denervation abolished differences in urinary water and sodium excretion and RBF between conscious and sevoflurane anesthetized sheep. The findings support the hypothesis that the volatile anesthetic sevoflurane causes an increase in RSNA, which results in renin release with little or no influence on AVP release. Together these effects reduce RBF and enhance renal tubular water and sodium reabsorption leading to retention of water and sodium. Moreover, the excitatory or disinhibitory effect of sevoflurane on RSNA remains, although briefly blunted, despite substantial plasma volume expansion, which may explain why fluid resuscitation during anesthesia in many patients is ineffective in improving urine output and preventing postoperative AKI.

Adequate fluid balance is crucial to protect organ function in the perioperative period.^27^ Fluid therapy has been described as a U-shaped curve where a too liberal or a too restrictive fluid therapy has negative consequences.^28^ Norberg et al showed in healthy volunteers that isoflurane induced retention of water and increased circulating levels of renin and aldosterone, and to a much lesser extent, vasopressin.^29^ We could not demonstrate a significant change in AVP in sevoflurane anesthetized surgical patients compared with when they were conscious. This may be related to the fasting regimen applied which allowed the children to drink freely up until 2 h prior to anesthesia, preventing major dehydration. However, renin was increased almost 10-fold compared with the conscious state in association with decreased urinary water and sodium excretion. Hypotension and hypovolemia result in unloading of cardiovascular baroreceptors that potentially leads to increased RSNA and reduced GFR, which, along with decreased arterial pressure, are main stimuli for renin release.^24,30^ Renin is stored in granules of juxtaglomerular cells (JGC) located near the vascular pole of the glomerulus.^31^ Specialized epithelial cells in the cortical thick ascending limb called the macula densa sense the urinary NaCl. A reduction in macula densa NaCl-transport is interpreted as an indication of lowered GFR, which stimulates the release of renin from the juxtaglomerular apparatus (JGA).^32^ RSNA increases circulating levels of renin through beta-adrenergic receptors on the JGCs.^33^ Anesthesia may induce cardiovascular instability but hypotension was not evident in sheep or children and subjects were properly fluid loaded to prevent hypovolemia regardless of study protocol. After volume expansion in sheep creatinine-clearance was similar with and without sevoflurane. The finding that RSNA remained significantly elevated even after a substantial fluid load in anesthetized sheep and that ARB had no additional effects beyond renal denervation on RBF suggests that RSNA and not decreased GFR is the most important mediator of renin release during sevoflurane anesthesia. However, since creatinine-clearance was decreased by sevoflurane in the pediatric experiments (where p-renin was measured) firm conclusions regarding increased RSNA and decreased GFR as the major stimuli for renin release during anesthesia cannot be drawn from these experiments.

Sevoflurane is considered superior to most inhaled anesthetics because of its rapid onset and recovery in combination with little airway irritation.^34^ Briefly it potentiates central nervous system gamma-aminobutyric acid receptor type A (GABA)-receptor function causing anesthesia.^35^ The effect of volatile anesthetics on RSNA in man is unknown since RSNA cannot be directly measured in humans. Indirect evidence indicates that volatile anesthesia decreases total sympathetic nerve activity in man as suggested by the finding of reduced plasma noradrenaline levels.^36,37^ Sevoflurane also attenuates baroreflex function and may inhibit the peripheral sympathetic nervous system.^36,38^ Human muscle sympathetic nerve activity (MSNA) has shown to be either unaffected or decreased by volatile anesthesia depending on the depth of anesthesia,^39^ and skin sympathetic nerve activity is depressed by numerous anesthetics.^40^ These conflicting results are likely to depend on various factors, including the experimental setting, but one reason may be that anesthesia has independent and contrasting effects on sympathetic outflow to different organs. In support of this hypothesis are the results in cats that anesthesia induces greater and longer lasting reductions in cardiac sympathetic nerve activity (CSNA) than RSNA.^41^ We have also found, in sheep, that isoflurane anesthesia completely abolishes CSNA, but does not inhibit RSNA (previous unpublished findings). Moreover, strong evidence exists that distinct populations of sympathetic premotor neuron in separate regions of the brainstem and hypothalamus control sympathetic outflow to different organs.^42,43^ This may contribute to differential responses in CSNA and RSNA in response to fluid loading.^44^ As described here, RSNA was tonically increased by sevoflurane and only showed a small and transient decrease in response to fluid loading. This may be due to stimulation of RSNA by sevoflurane but since GABA generally acts as an inhibitory neurotransmitter in the sympathetic nervous system^45^ it is also plausible that sevoflurane attenuates a tonic inhibition of RSNA, ultimately resulting in increased RSNA.

RSNA causes the release of renin from granular cells in the juxtaglomerular apparatus.^24^ Subsequently this leads to the formation of angiotensin II (AngII) and release of aldosterone.^24,46^ RSNA, AngII, and aldosterone all promote renal water and sodium reabsorption via direct tubular effects.^46,47^ In agreement with our hypothesis increased RSNA may thus largely explain the reduction in water and sodium excretion by sevoflurane, as further corroborated by the finding that renal denervation completely reversed this effect. AVP or macula densa mediated renin release appear less important, at least in the current setting. This was suggested previously ^48^ where unilateral renal denervation in the dog resulted in elevated RBF, GFR, and sodium excretion in the denervated kidney during anesthesia. Increased sodium excretion in response to renal denervation has also been demonstrated in hypertensive humans ^49^ and rats.^50^ It is likely that this effect is more pronounced in settings where RSNA is elevated which may explain our finding of increased sodium excretion by renal denervation in the sevoflurane-anesthetized group but not in the conscious animals. Following bilateral renal denervation the natriuretic response to fluid loading was augmented in anesthetized sheep. This is in line with the hypothesis that continuously elevated RSNA due to sevoflurane regardless of plasma volume status causes sustained antinatriuresis. Renal denervation in conscious sheep had no significant effect on the natriuretic response to a fluid load. It has previously been demonstrated in sheep that chronic renal denervation attenuates the natriuretic response to saline infusion.^51^ The reason for the discrepancy with the current study is not clear but may potentially be related to differences in volume status at baseline, tonicity of infused solution or baseline level of RSNA. The latter is supported by the fact that natriuresis is blunted in volume loaded hypertensive rats but not in normotensive rats.^52^

AKI is a frequent postoperative complication and an independent risk factor for mortality and morbidity.^53,54^ The causes of postoperative AKI are largely unknown but ischemia is likely a common trigger.^55^ The volatile anesthetic isoflurane reduces RBF in man by approximately 50%.^56^ Prior experimental data in sheep where RBF was measured directly also show a drastic reduction with isoflurane ^26^ and to a lesser extent with propofol-anesthesia.^16^ The reduction in RBF by isoflurane is associated with renal hypoxia and elevated RSNA.^11,16^ Consistent with the effects of isoflurane we demonstrate that sevoflurane also reduces RBF and increases RSNA. Renal denervation normalized RBF indicating that at least part of the reduction in RBF during sevoflurane anesthesia is due to RSNA-induced renal vasoconstriction. Anesthesia with isoflurane ^57^ or propofol ^58^ reduce renal oxygen consumption in sheep. In theory an elevation in RSNA by sevoflurane may increase renal oxygen consumption by stimulating excessive renal tubular sodium reabsorption and reduce renal oxygen delivery by renal vasoconstriction. This potentially confers an increased risk of AKI by sevoflurane compared with other anesthetics in susceptible patients, as supported by reports of higher incidence and severity of renal failure after sevoflurane compared with propofol anesthesia,^59^ but further studies are needed to confirm this conclusion. It is well known that AngII is a potent renal vasoconstrictor and enhanced RBF and improved renal oxygenation by pharmacological angiotensin-II receptor type 1 blockade have been described in sheep, pigs and rats in various experimental settings.^60–62^ Our results did not demonstrate a significant (*P* = .16) increase in RBF by losartan in sheep under sevoflurane anesthesia. This may be due to lack of statistical power but could also be the result of renal denervation and major fluid loading reducing the circulating levels of AngII.

Intravenous volume loading by isotonic fluids is a measure regularly performed by clinicians to counteract reduced urinary output and prevent assumed renal hypoperfusion.^63^ Oliguria under anesthesia may predict AKI,^7,64^ but the effect of a fluid load on urinary output is highly variable. Moreover, striving toward a predefined urine output target to prevent AKI is futile.^65,66^ Our results form a physiological basis for these findings as RSNA remained high during anesthesia even after a significant fluid load. Volume loading was also unable to improve RBF under sevoflurane anesthesia. Given the caveat that our sheep were well hydrated to start with this suggests that heightened stimulation of RSNA during sevoflurane anesthesia attenuates the beneficial effect of goal directed fluid therapy on RBF, diuresis and natriuresis.

## Limitations

To identify true RSNA signal from background noise we made recordings of minimal RSNA as established with infusion of phenylephrine to saturate high pressure baroreceptors. In this manner, zeroRSNA was calculated. We were only able to make one calculation of zeroRSNA for each of the experiments, at the onset of the experiment. Errors may have increased with longer time intervals between the calculation of background noise and the actual time of various recordings. We are not able to measure RSNA in man, and extrapolating findings from animal studies to human physiology is not uncomplicated. Previous reports on anesthesia and its influence on sympathetic nerve activity (SNA) seem to differ among different species. This may be due to actual differences between species, different study protocols, and differential central control of SNA.^16,43^ The human study was performed on children, and further studies are needed to explore whether the findings are applicable to adults as well. The pediatric surgery study included a sacral epidural single injection analgesia, but this has been demonstrated not to influence kidney function.^67^

We had small numbers of animals but by using them as their own controls we were able to obtain sufficient statistical power. Our focus was RSNA and its potential renal effects. Therefore, we did not attempt to measure sympathetic nerve activity in systemic regions other than the kidney.

## Conclusion

The reason volatile anesthesia results in oliguria is generally conceived to be due to excessive AVP-release or hypotension. In experimental and human studies we have found compelling evidence that increased RSNA is the major cause of decreased urinary water and sodium excretion and RBF during normotensive sevoflurane anesthesia. Besides providing a novel mechanistic explanation to why urine output is decreased by volatile anesthetics, these findings may be considered when choosing an anesthetic method. Especially in patients at risk for ischemic AKI. Known pharmacological agents that diminish the effects of increases in RSNA and angiotensin II on the kidney could be explored for their effect on surgical outcomes.

## Contributions

Conceptualization and design: MT and RF; methodology and data collection: all authors; data analysis: MT and RF; writing—original draft preparation, MT; writing—review and editing, all authors; supervision: RF and GFD; and funding acquisition: RF.

## Supplementary Material

zqab042_Supplementary_tables_and_figure_legends

## Data Availability

The data underlying this article will be shared on reasonable request to the corresponding author.
