# Supplementary material for: Role of Renal Sympathetic Nerve Activity in Volatile Anesthesia's Effect on Renal Excretory Function
Source: Function (Oxf). 2021 Aug 20;2(6):zqab042. doi: 10.1093/function/zqab042 (PMC8788708; doi:10.1093/function/zqab042)
Supplement: zqab042_Supplementary_tables_and_figure_legends [file zqab042_supplementary_tables_and_figure_legends.docx]

| **mean** |  |  | |  | |  | |
| --- | --- | --- | --- | --- | --- | --- | --- |
| Parameter | Conscious (mean) | (SD) | | Sevoflurane (mean) | | (SD) | |
| **RR** (breaths per minute) | | | |  | |  | |
| Baseline | N/A |  | | 22.86 | | 4.67 | |
| Fluid +2h | N/A |  | | 20.57 | | 4.72 | |
| **CO** (L/min) | |  | |  | |  | |
| Baseline | 4.99 | 0.72 | | 3.99 | | 0.52 | |
| Fluid +2h | 6.04 | 1.23 | | 5.18 | | 1.16 | |
| **CVP** (mmHg) | |  | |  | |  | |
| Baseline | 1.96 | 0.64 | | 1.93 | | 0.53 | |
| Fluid +2h | 3.17 | 1.82 | | 2.06 | | 0.84 | |
| **EtCO2** (kPa) | |  | |  | |  | |
| Baseline | N/A |  | | 5.33 | | 0.28 | |
| Fluid +2h | N/A |  | | 5.19 | | 0.23 | |
| **EtO2** (kPa) | |  | |  | |  | |
| Baseline | N/A |  | | 31.71 | | 4.15 | |
| Fluid +2h | N/A |  | | 32.00 | | 1.91 | |
| **FiO2** (%) |  |  | |  | |  | |
| Baseline | 21.00 | 0.00 | | 35.43 | | 4.65 | |
| Fluid +2h | 21.00 | 0.00 | | 37.14 | | 1.46 | |
| **HR** (beats per min) | | | |  | |  | |
| Baseline | 77.86 | 10.75 | | 96.14 | | 14.02 | |
| Fluid +2h | 71.14 | 12.90 | | 92.86 | | 10.48 | |
| **MAP** (mmHg) | |  | |  | |  | |
| Baseline | 95.00 | 5.23 | | 104.14 | | 9.46 | |
| Fluid +2h | 100.14 | 7.86 | | 96.43 | | 13.28 | |
| **PA** (mmHg) | |  | |  | |  | |
| Baseline | 15.29 | 5.25 | | 18.86 | | 4.56 | |
| Fluid +2h | 14.86 | 4.67 | | 17.33 | | 3.27 | |
| **PCWP** (mmHg) | |  | |  | |  | |
| Baseline | 4.43 | 2.51 | | 4.29 | | 2.56 | |
| Fluid +2h | 8.17 | 2.79 | | 5.50 | | 2.43 | |
| **RBF** (mL/min) | |  | |  | |  | |
| Baseline | 314.40 | 65.46 | | 191.44 | | 68.65 | |
| Fluid +2h | 326.00 | 70.07 | | 205.30 | | 62.72 | |
| **Temp** (°C) |  |  | |  | |  | |
| Baseline | 39.29 | 0.47 | | 39.30 | | 0.70 | |
| Fluid +2h | 39.14 | 0.20 | | 39.49 | | 0.74 | |
| **TV** (mL) |  |  | |  | |  | |
| Baseline | N/A |  | | 358.57 | | 57.28 | |
| Fluid +2h | N/A |  | | 375.71 | | 79.55 | |
| **RVR** (mmHg/mL/min) | | | |  | |  | |
| Baseline | 0.31 | 0.09 | | 0.60 | | 0.23 | |
| Fluid +2h | 0.31 | 0.07 | | 0.49 | | 0.14 | |
| **TPR (**mmHg/mL/min) | | |  | |  | |  |
| Baseline | 19.10 | 3.60 | | 26.09 | | 4.83 | |
| Fluid +2h | 16.59 | 3.38 | | 18.63 | | 2.94 | |

Supplemental table 1 **Hemodynamic, respiratory and temperature parameters at baseline and 2 hours after fluid loading in conscious or sevoflurane anesthetized sheep**

Data are expressed as means and standard deviation at baseline and 2 hours after fluid loading (Ringer’s Acetate, 20 ml/kg in 30 min). n = 7 with repeated measurements in the same sheep conscious and during sevoflurane anesthesia. RR = respiratory rate. CO = cardiac output. CVP = central venous pressure. EtCO2 = end tidal CO2. EtO2 = end tidal O2. FiO2 = percent oxygen in breathing air, HR = heart rate. MAP = mean arterial pressure. PA = pulmonary artery pressure. PCWP = pulmonary capillary wedge pressure. RBF = renal blood flow. Temp = core temperature. TV = tidal volume. RVR = renal vascular resistance is the quotient of (MAP-CVP) divided by RBF. TPR = total peripheral resistance is the quotient of (MAP-CVP) divided by CO. N/A = Not Available, not measured

| **mean** |  |  |  |  |  |  |  |  |
| --- | --- | --- | --- | --- | --- | --- | --- | --- |
| Parameter | Consc RDN (mean) | (SD) | Sevo RDN (mean) | (SD) | DS AVP-(mean)i | (SD) | DS los mean | ()SD |
| **RR** (breaths per minute) | |  |  |  |  |  |  |  |
| Baseline | N/A |  | 14.86 | 0.90 | 13.86 | 1.07 | 15.71 | 2.63 |
| Fluid +2h | N/A |  | 15.43 | 1.40 | 14.00 | 2.08 | 15.57 | 2.30 |
| **CO** (L/min) | |  |  |  |  |  |  |  |
| Baseline | 6.69 | 1.44 | 5.61 | 0.94 | 5.28 | 0.83 | 4.65 | 0.87 |
| Fluid +2h | 7.51 | 1.35 | 6.19 | 0.91 | 5.36 | 1.34 | 5.66 | 0.88 |
| **CVP** (mmHg) | |  |  |  |  |  |  |  |
| Baseline | 3.71 | 2.72 | 2.21 | 2.81 | 1.29 | 1.29 | 1.36 | 1.49 |
| Fluid +2h | 3.79 | 1.82 | 2.79 | 4.20 | 1.07 | 1.17 | 1.24 | 0.84 |
| **EtCO2** (kPa) | |  |  |  |  |  |  |  |
| Baseline | N/A |  | 5.47 | 0.39 | 5.73 | 0.50 | 5.36 | 0.48 |
| Fluid +2h | N/A |  | 5.89 | 0.24 | 5.73 | 0.36 | 5.76 | 0.41 |
| **EtO2 (**kPa) | |  |  |  |  |  |  |  |
| Baseline | N/A |  | 36.57 | 7.35 | 36.86 | 6.36 | 34.57 | 5.38 |
| Fluid +2h | N/A |  | 34.00 | 8.54 | 33.57 | 4.08 | 31.57 | 5.53 |
| **FiO2** (%) | |  |  |  |  |  |  |  |
| Baseline | 21.00 | 0.00 | 43.00 | 6.58 | 42.86 | 5.87 | 40.43 | 5.00 |
| Fluid +2h | 21.00 | 0.00 | 41.00 | 8.14 | 38.71 | 5.85 | 38.00 | 5.54 |
| **HR** (beats per min) | |  |  |  |  |  |  |  |
| Baseline | 74.43 | 13.23 | 101.57 | 17.75 | 104.14 | 28.68 | 105.00 | 11.24 |
| Fluid +2h | 69.29 | 6.90 | 109.29 | 15.15 | 107.71 | 25.57 | 112.00 | 15.56 |
| **MAP** (mmHg) | |  |  |  |  |  |  |  |
| Baseline | 96.43 | 13.84 | 108.71 | 11.93 | 116.57 | 17.52 | 110.00 | 13.53 |
| Fluid +2h | 107.43 | 16.82 | 115.00 | 9.70 | 110.86 | 14.74 | 113.43 | 14.18 |
| **PA** (mmHg) | |  |  |  |  |  |  |  |
| Baseline | 16.43 | 5.86 | 13.86 | 1.46 | 15.86 | 4.22 | 19.14 | 7.73 |
| Fluid +2h | 20.43 | 3.60 | 17.43 | 2.64 | 14.14 | 2.19 | 16.71 | 2.29 |
| **PCWP** (mmHg) | |  |  |  |  |  |  |  |
| Baseline | 6.67 | 1.37 | 8.17 | 1.72 | 7.60 | 2.97 | 8.80 | 1.48 |
| Fluid +2h | 8.60 | 1.52 | 8.00 | 1.15 | 7.33 | 2.08 | 9.00 | 1.41 |
| **RBF** (mL/min) | |  |  |  |  |  |  |  |
| Baseline | 296.67 | 85.11 | 254.33 | 16.33 | 206.50 | 30.62 | 215.50 | 52.32 |
| Fluid +2h | 307.05 | 58.60 | 278.73 | 32.32 | 256.25 | 57.78 | 248.25 | 34.85 |
| **Temp** (°C) |  |  |  |  |  |  |  |  |
| Baseline | 39.40 | 0.40 | 37.97 | 0.50 | 38.64 | 0.38 | 38.34 | 0.43 |
| Fluid +2h | 39.87 | 0.66 | 38.40 | 0.75 | 39.09 | 0.53 | 38.73 | 0.38 |
| **TV** (mL) | |  |  |  |  |  |  |  |
| Baseline | N/A |  | 570.00 | 22.36 | 507.14 | 34.98 | 540.00 | 27.08 |
| Fluid +2h | N/A |  | 565.71 | 20.70 | 510.00 | 35.12 | 540.00 | 27.08 |
| **RVR (**mmHg/mL/min) | |  |  |  |  |  |  |  |
| Baseline | 0.32 | 0.09 | 0.41 | 0.07 | 0.54 | 0.10 | 0.54 | 0.12 |
| Fluid +2h | 0.33 | 0.08 | 0.36 | 0.07 | 0.43 | 0.12 | 0.45 | 0.06 |
| **TPR** (mmHg/mL/min) | |  |  |  |  |  |  |  |
| Baseline | 14.59 | 4.49 | 19.37 | 3.82 | 23.28 | 5.33 | 23.68 | 5.05 |
| Fluid +2h | 14.65 | 5.85 | 17.31 | 4.26 | 21.37 | 5.00 | 20.24 | 3.76 |

Supplemental table 2 **Hemodynamic, respiratory and temperature parameters at baseline and 2 hours after fluid loading in conscious or sevoflurane anesthetized sheep with renal denervation**

Data are expressed as means and standard deviation at baseline and 2 hours after fluid loading (Ringer’s Acetate, 20 ml/kg over 30 min). n = 7 with repeated measurements in the same sheep conscious and during sevoflurane anesthesia. RR = respiratory rate. CO = cardiac output. CVP = central venous pressure. EtCO2 = end tidal CO2. EtO2 = end tidal O2. FiO2 = percent oxygen in breathing air, HR = heart rate. MAP = mean arterial pressure. PA = pulmonary artery pressure. PCWP = pulmonary capillary wedge pressure. RBF = renal blood flow. Temp = core temperature. TV = tidal volume RVR = renal vascular resistance is the quotient of (MAP-CVP) divided by RBF. TPR = total peripheral resistance is the quotient of (MAP-CVP) divided by CO. N/A = Not Available, not measured. Consc RDN = conscious, renal denervation. Sevo RDN = sevoflurane, renal denervation. DS AVP-i = renal denervation, sevoflurane anesthesia and AVP-inhibitor. DS los = renal denervation, sevoflurane anesthesia and losartan (angiotensin receptor blocker).

Supplemental table 3: Changes in physiological parameters between baseline and 2 hours after start of fluid infusion (Ringer’s Acetate, 20 ml/kg over 30 min) expressed as means and standard deviation of differences between post infusion and baseline. Renal denervated sheep (n=7) conscious and during sevoflurane anesthesia compared with sheep with intact nerves (n=7) conscious and during sevoflurane anesthesia. FENa = fractional excretion of sodium, SD = standard deviation, sevo = sevoflurane, RDN = bilateral renal denervation, los = angiotensin II antagonist losartan, AVP-i = arginine-vasopressin inhibitor.


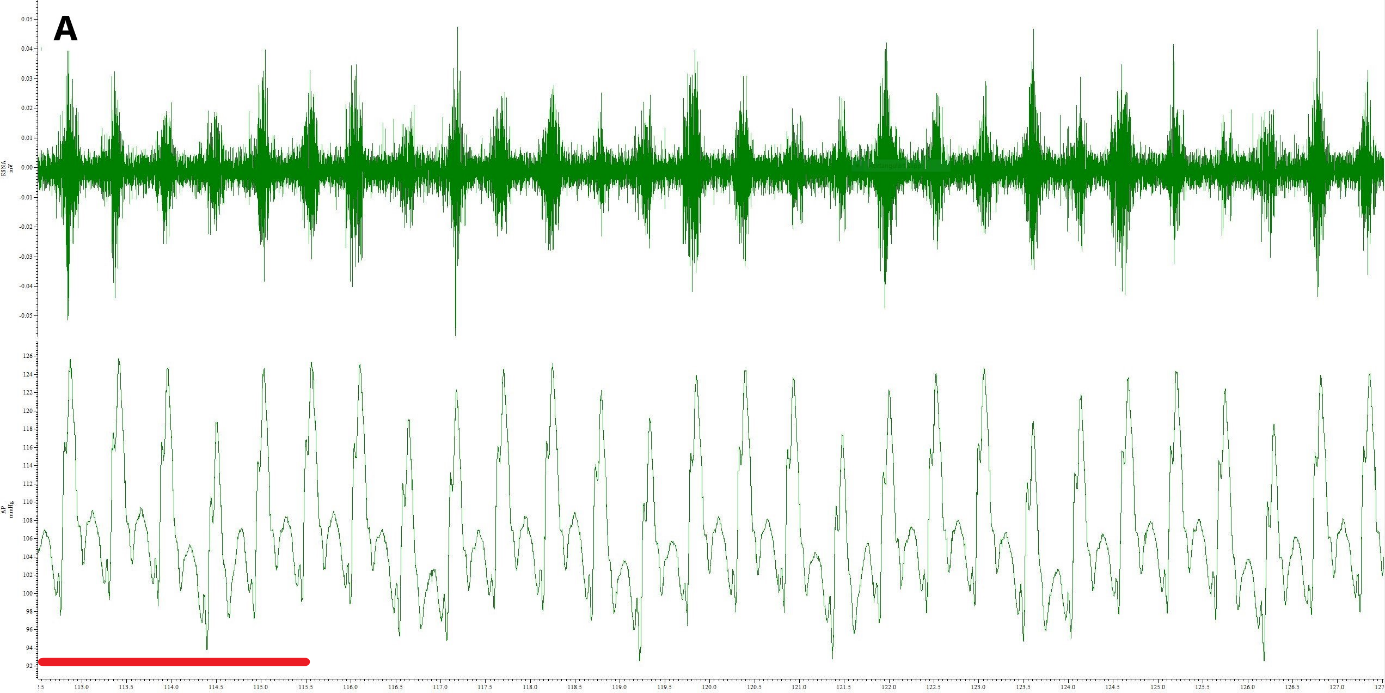


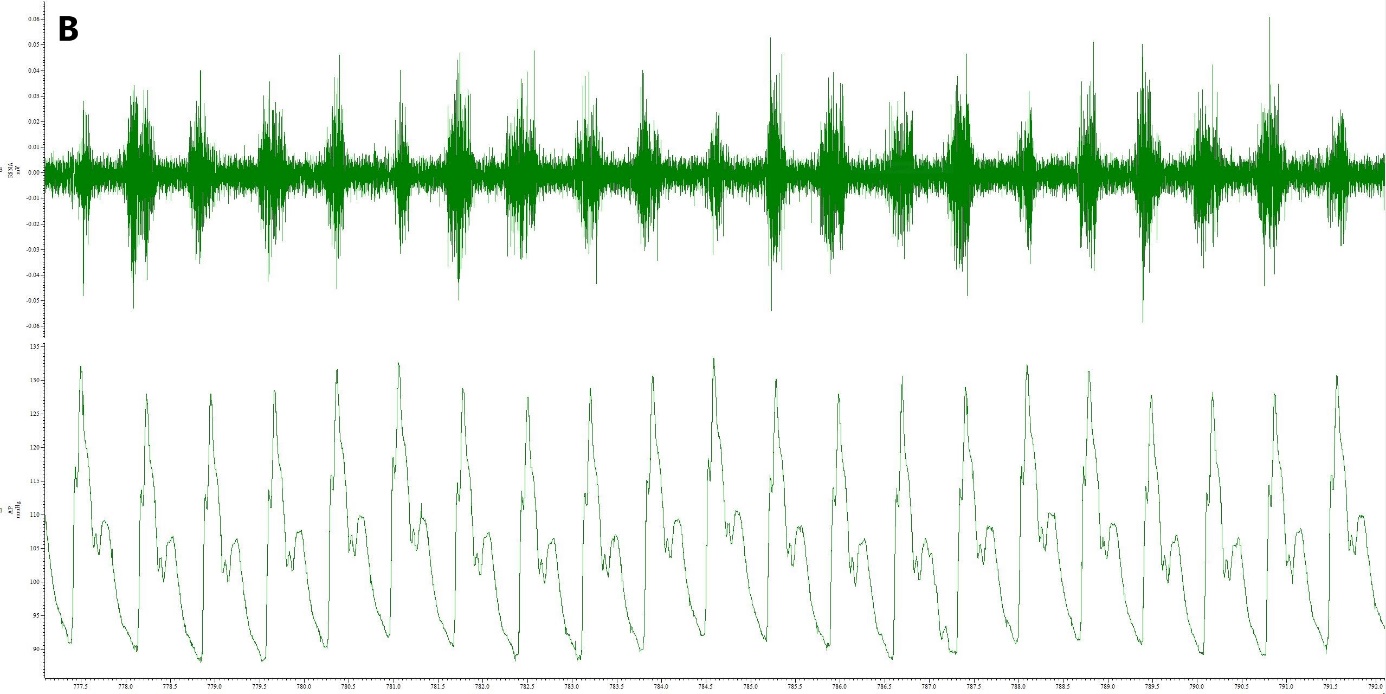


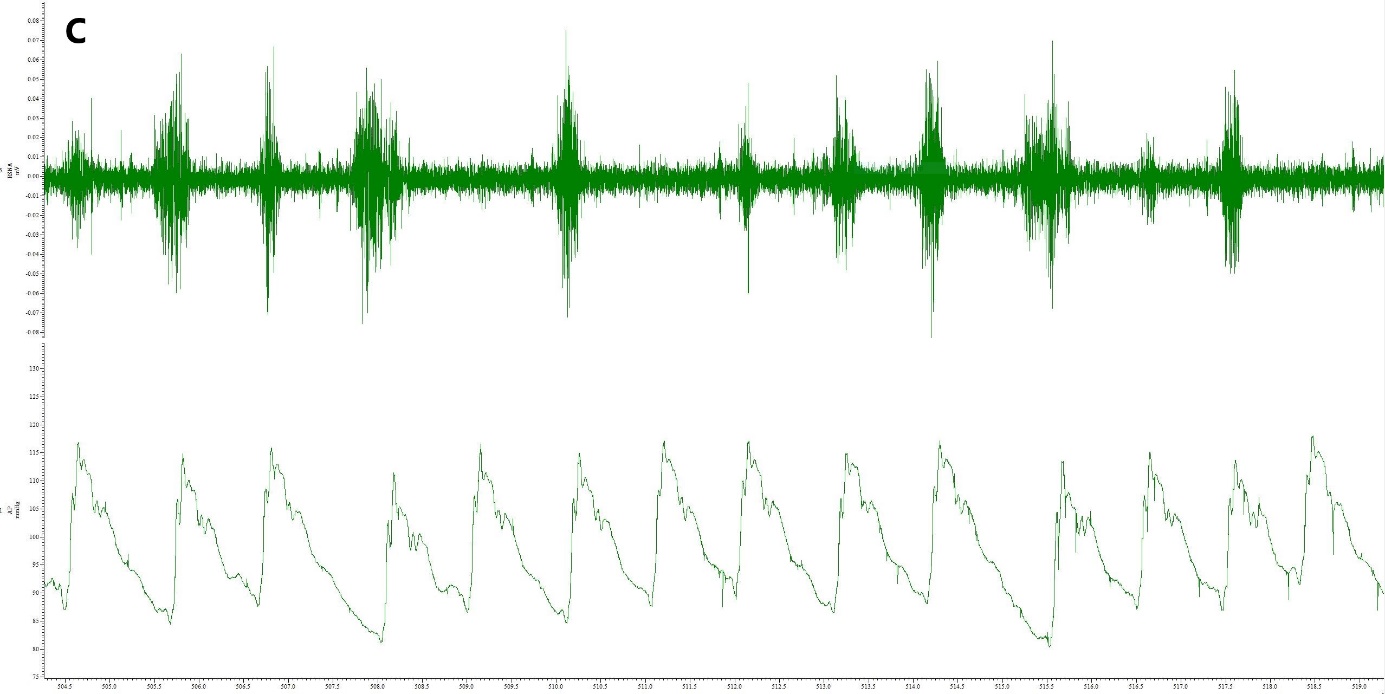


Supplemental figure 1: Original recording of RSNA (upper trace in all three images) and AP (lower trace in all three images). A and B from the same experiment (sevoflurane anesthesia) and C same animal in conscious experiment. A and C are spontaneous breathing and B is mechanical ventilation. Burst: heartbeat ratio 1:1 in A and B, but 5:7 in C. All are 15 seconds recordings. The red line in A for scale represents three seconds.


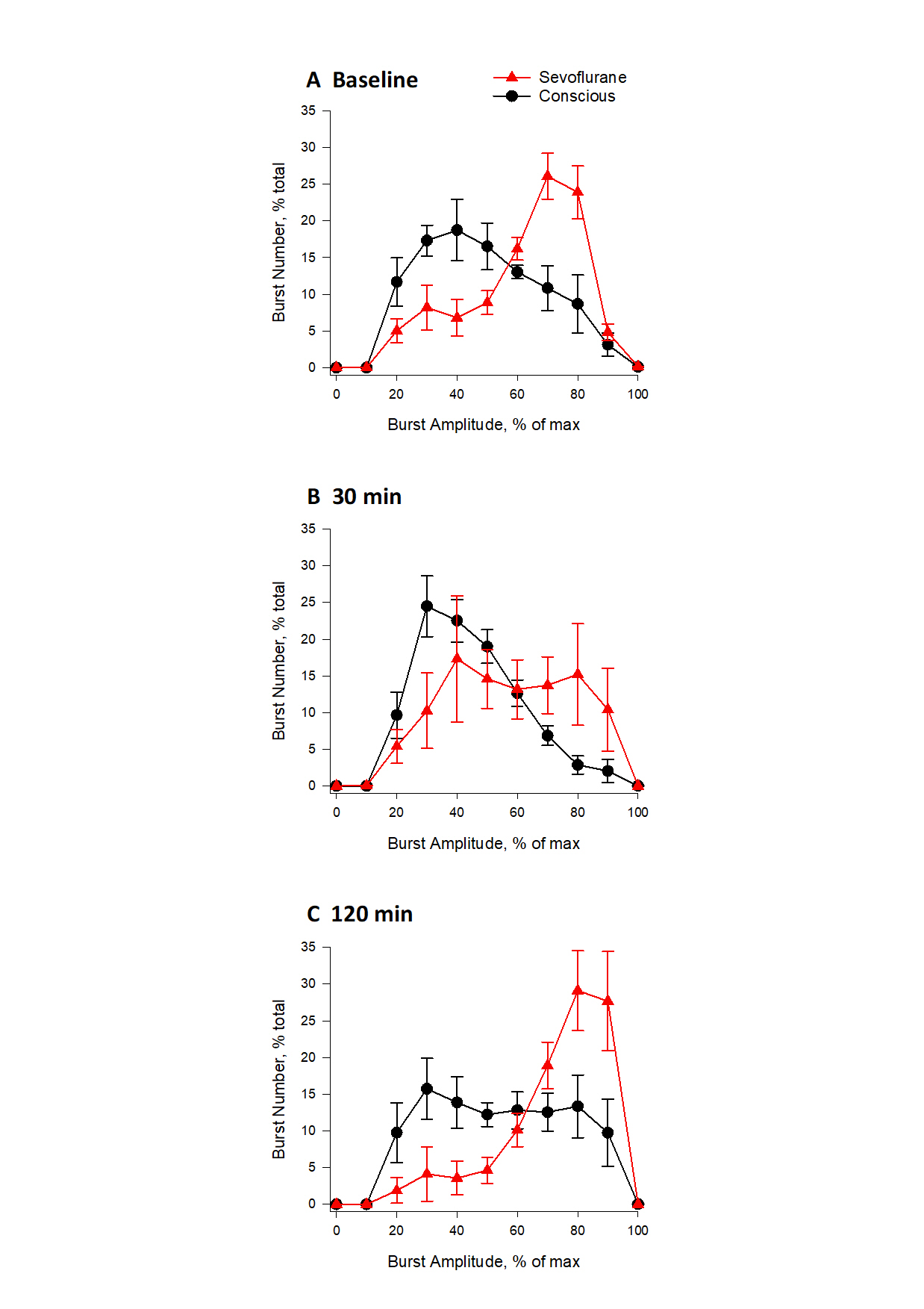


Supplemental figure 2: Frequency distribution of burst amplitude in conscious and sevoflurane-anesthetized sheep. The burst frequency was calculated as the number of bursts per minute. Burst amplitude was calculated as the integrated area under the curve for each burst. The largest burst during baseline recordings for each sheep was used as reference and the sizes of all remaining bursts were calculated as a percentage of this burst. The relative burst amplitudes were calculated by determining the frequency distribution of all the bursts during the baseline period, and at 30 min and 120 min after commencing fluid loading with Ringer’s Acetate.
